# Feasibility, outcomes and follow-up analysis of transcatheter closure of outlet ventricular septal defect with various devices from North-Eastern India: a single centre observational study

**DOI:** 10.1186/s43044-026-00719-6

**Published:** 2026-02-27

**Authors:** Saurabhi Das, Shantanu Jain, Nayem Raja, Narendra Sharma

**Affiliations:** Department of Pediatric Cardiology, Health City Hospital , Khanapara, Assam Guwahati, India

**Keywords:** Ventricular septal defect (VSD), Transcatheter, Aortic regurgitation (AR)

## Abstract

**Background:**

Outlet septum ventricular septal defects (VSDs) are notably more prevalent in North-Eastern India and often associated with aortic valve prolapse, leading to a need for surgical intervention. While surgical methods remain the conventional approach, there is an opportunity to explore the effectiveness of transcatheter closure techniques.

**Objective and Methodology:**

This study aimed to assess the effectiveness and follow-up of transcatheter closure for outlet and outlet muscular type VSDs using three distinct devices: Amplatzer ADO II, KONAR MF VSD Occluder, and Cocoon VSD Occluder. This Descriptive study with follow-up observation up to 1 year was conducted at Health City Hospital in Guwahati, India, from March 2023 to November 2025, involving 21 patients who met the criteria for inclusion.

**Results:**

Among the 21 patients, transcatheter closure was successfully achieved in 16 individuals, resulting in a success rate of 76.2%. The mean diameter of the VSDs was 3.8 mm, with 66.6% of the cases showing pre-existing aortic valve prolapse. Closure attempts were unsuccessful in five patients, who subsequently received surgical intervention. In the successful cases, the Amplatzer ADO II was implanted in 7 patients (43.7%), the KONAR MFO in 8 patients (50%), and the Cocoon VSD occluder in 1 patient (6.3%). Seven patients exhibited mild intra-device residual shunts, and 37.5% (6/16) experienced a transient increase in aortic regurgitation (AR) after 24 hours post-procedure. Importantly, follow-up after one year showed static or non-progression of pre-existing AR, with worsening of AR (moderate severity) observed only in one patient, and the majority of residual shunts resolved. No major complications like outflow obstruction or device embolization were reported in the present study.

**Conclusion:**

The results indicate that transcatheter closure of outlet and outlet muscular type VSDs is a promising option for selected patients, yielding positive short- to mid-term outcomes. Further long-term follow-up will enhance our understanding of the procedure’s safety and efficacy, paving the way for broader application of this technique in clinical practice.

**Supplementary Information:**

The online version contains supplementary material available at 10.1186/s43044-026-00719-6.

## Introduction

Outlet septum VSDs are situated just below the semilunar valves in the outlet septum, in proximity to the aortic and pulmonary valves [1–2]. These defects are frequently associated with varying degrees of aortic valve prolapse, which can progress due to the Venturi effect, potentially leading to distortion and an increased incidence of aortic regurgitation. Surgical repair is the primary method for treating these types of VSDs and has demonstrated a high success rate. In some selected cases of outlet septum defects, there may be a muscular conal margin between the aortic and pulmonary valves, where transcatheter closure has yielded acceptable outcomes comparable to published surgical series [3]. This study assesses the feasibility and outcomes of transcatheter closure of outlet and outlet muscular VSDs complicated by aortic valve prolapse, with or without aortic regurgitation, using three different devices in both children and adults.

## Methodology

### Study design

This Descriptive observational study was carried out in the Division of Paediatric Cardiology in Health City Hospital, Guwahati, Assam, India. Retrospectively, data of 21 patients who underwent transcatheter closure of VSD (outlet and outlet muscular) from March 2023 to November 2024 were collected, and a prospective observation of successful cases was conducted up to 1 year. No formal hypothesis testing was done in the present study due to the small sample size.

### Case selection criteria

VSD (outlet and outlet muscular) closure is indicated for patients with the following inclusion criteria.

1. All outlet and outlet muscular VSD with at least 1–2 mm aortic rim,
2. Symptomatic or evidence of LA and LV enlargement in echocardiography.
3. With or without evidence of aortic cusp prolapse of equal or less than moderate severity.
4. Absence or presence of Aortic regurgitation equal to or less than mild in severity.
5. Defect size ≤ 8 mm.
6. Age > 1 year.
7. Written informed consent from guardians.

We included up to moderate aortic valve prolapse without significant AR cases, as closing the VSD can eliminate the “Venturi effect” that causes the prolapse and subsequent regurgitation, thus preventing further damage to the aortic valve.

In addition, patients having defect associated with gross cusp prolapse or cusp distortion or with ≥ moderate AR, presence of RV outflow obstruction or sub-aortic membrane, associated cardiac defects requiring cardiac surgery, defect size > 8 mm and irreversible pulmonary arterial hypertension were excluded from the study.

Any moderate AR severity patients were excluded upfront in the present study, as it is likely to worsen with device placement, and chronic friction can further damage the valve.

### Ethics

This study was approved by the Ethics Committees of Health City Hospital, Guwahati, India. All the research steps were performed according to the ethical standards and principles. Written informed consent was obtained from the parents or guardians of all patients included in the study.

### Statistical analysis

Categorical variables were expressed as numbers and percentages. Continuous variables were summarised as means. The statistical analyses were computed using the Statistical Package for the Social Sciences (SPSS Statistics), version 25. No inferential statistics were used.

## Definitions

### Outlet VSD (OVSD)

**Fig. 1:**
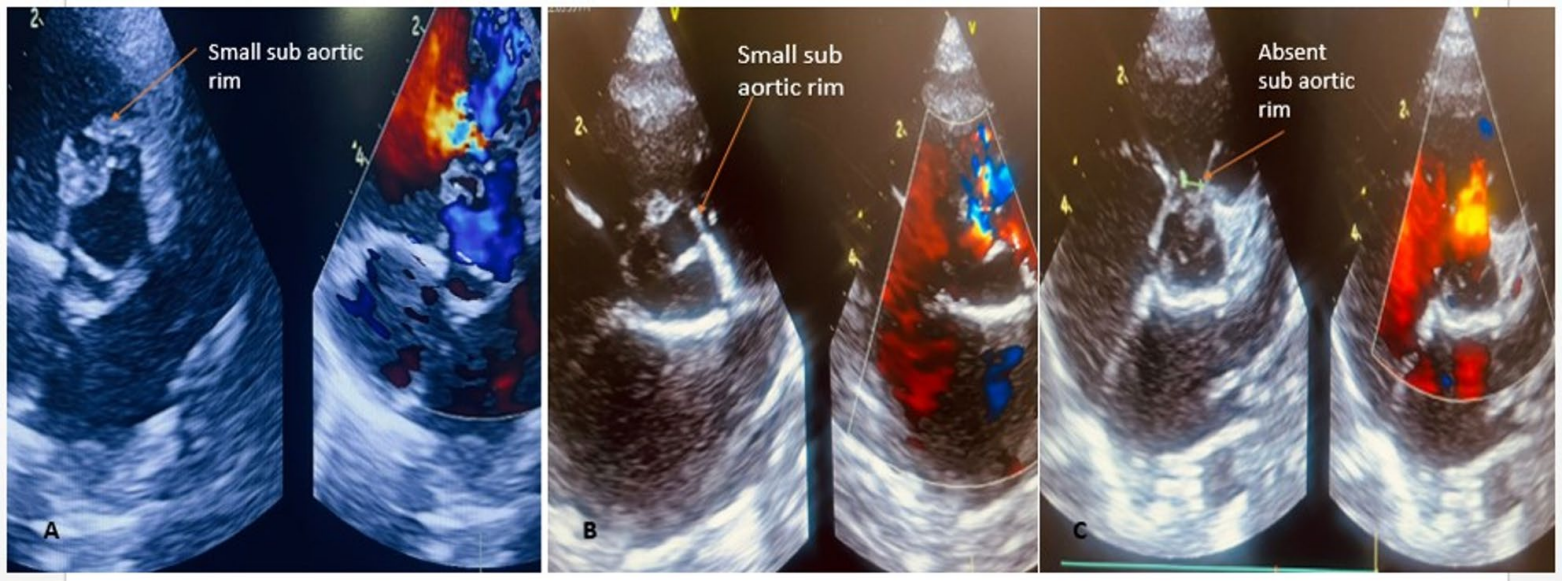
Echocardiographic profiling of outlet VSD in parasternal short-axis view, with **A**, **B** showing (arrow) presence of small subaortic rim in OVSD, **C** showing (arrow) an absent subaortic rim (not suitable for transcatheter closure)

The OVSD was defined by the defect of the interventricular septum with the presence of a colour flow jet around the 11–2 o’clock position in the parasternal short-axis view of TTE [3, 4] Fig. 1.

### Outlet muscular VSD (OMVSD)

**Fig. 2:**
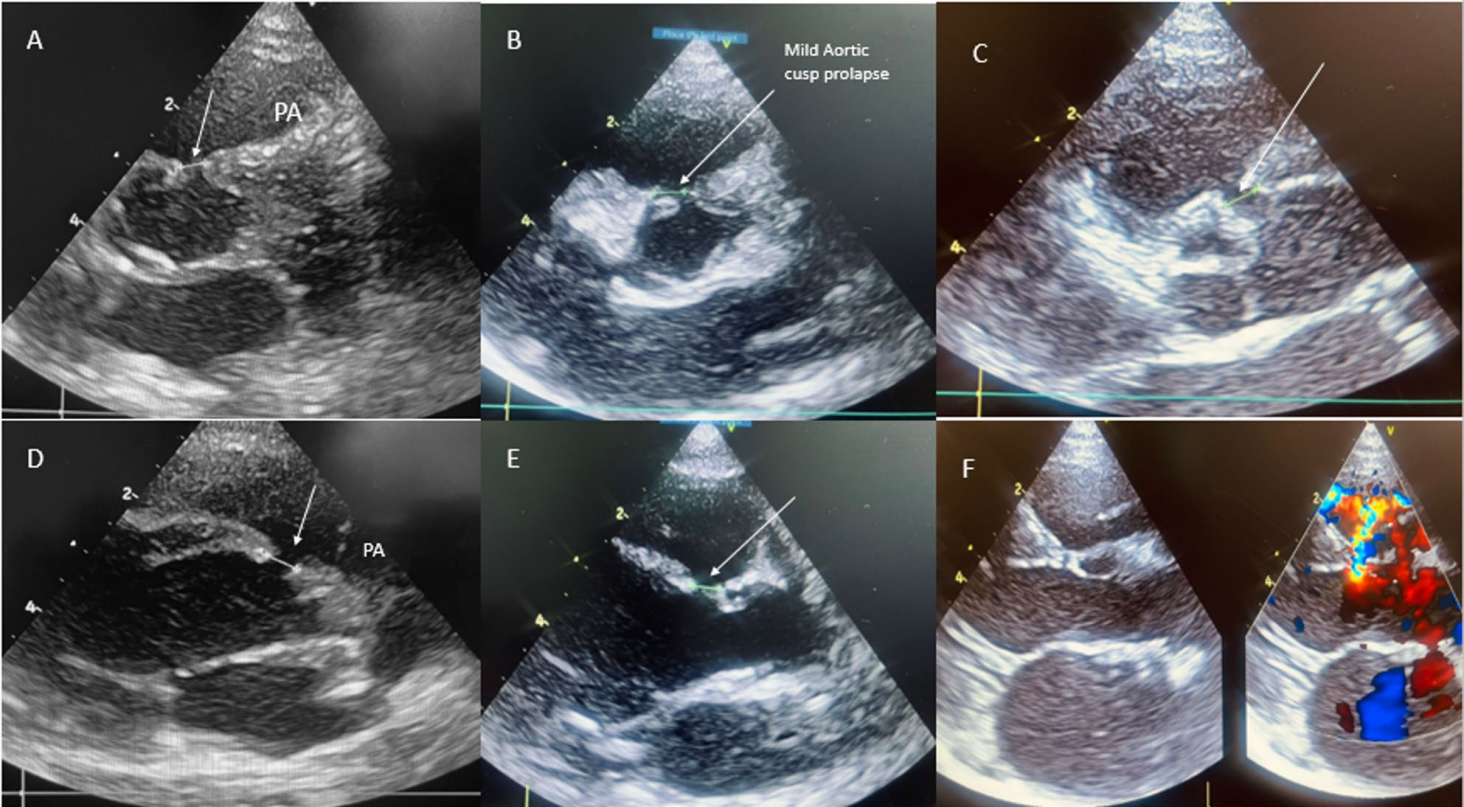
**A**-**E** showing modified parasternal short axis for complete profiling outlet muscular defect with varying degree of aortic valve prolapse and prominent muscular rim with arrow depicting defect and cusp prolapse, **F** showing outlet muscular defect in parasternal long axis view with colour Doppler interrogation

The OMVSD was defined by the defect of the interventricular septum with the presence of a colour flow jet around the 11–2 o’clock position in the posterior sweep from parasternal short-axis view of TTE, with the appearance of a muscular septum or defect not visible in the classic parasternal short-axis view (Fig. 2).

### Aortic valve prolapse (AVP)

 Aortic Valve prolapse [3–4] was graded a mild (buckling of the aortic cusp down the left ventricular [LV] outflow tract with minimal to no herniation into the VSD), moderate (prolapse of the cusp with obvious herniation and its sinus into the VSD); and severe (prolapse of the cusp and its sinus through the defect into the right ventricular [RV] outflow tract).

### Aortic regurgitation

The ratio of the jet width to the LVOT diameter was used to classify AR as (1) trivial: jet width/LVOT diameter < 10% AR jet visible in one echocardiographic view; (2) mild: jet width/LVOT diameter ≥ 10%–24%; (3) moderate: jet width/LVOT diameter ≥ 25%–49%; and (4) severe: jet width/LVOT diameter ≥ 50% [4].

Any AR if visible in only one plane of echocardiography was considered trivial in our study.

For better quantification, uniformity, and to avoid bias, we categorize trivial and mild AR degree in the same category as trivial -mild.

### Procedural technique

Medical records and all relevant documents of the patients were collected for clinical assessment, chest radiograph, electrocardiogram (ECG) and TTE at pre-procedure, during the procedure, immediate post-procedure, at 1–3 months, at 6 months, and 12 months post-procedure. The device position, presence of the residual shunt, and the severity of AVP and AR were assessed. The procedure time, length of hospital stays, and complications associated with the procedures were evaluated.

All patients received heparin (80 U/kg) before the procedure. Amoxicillin-clavulanic acid antibiotic, was administered at a dosage of 25 mg/kg. The initial diagnostic catheterization procedures were performed under anaesthesia in adults and conscious sedation in children. Vascular access was obtained through the right femoral artery and vein by using the modified Seldinger method. Complete right and left cardiac catheterization was performed, and documentation of pulmonary artery pressure, Qp: Qs, was done.

### VSD profiling and sizing

**Fig. 3:**
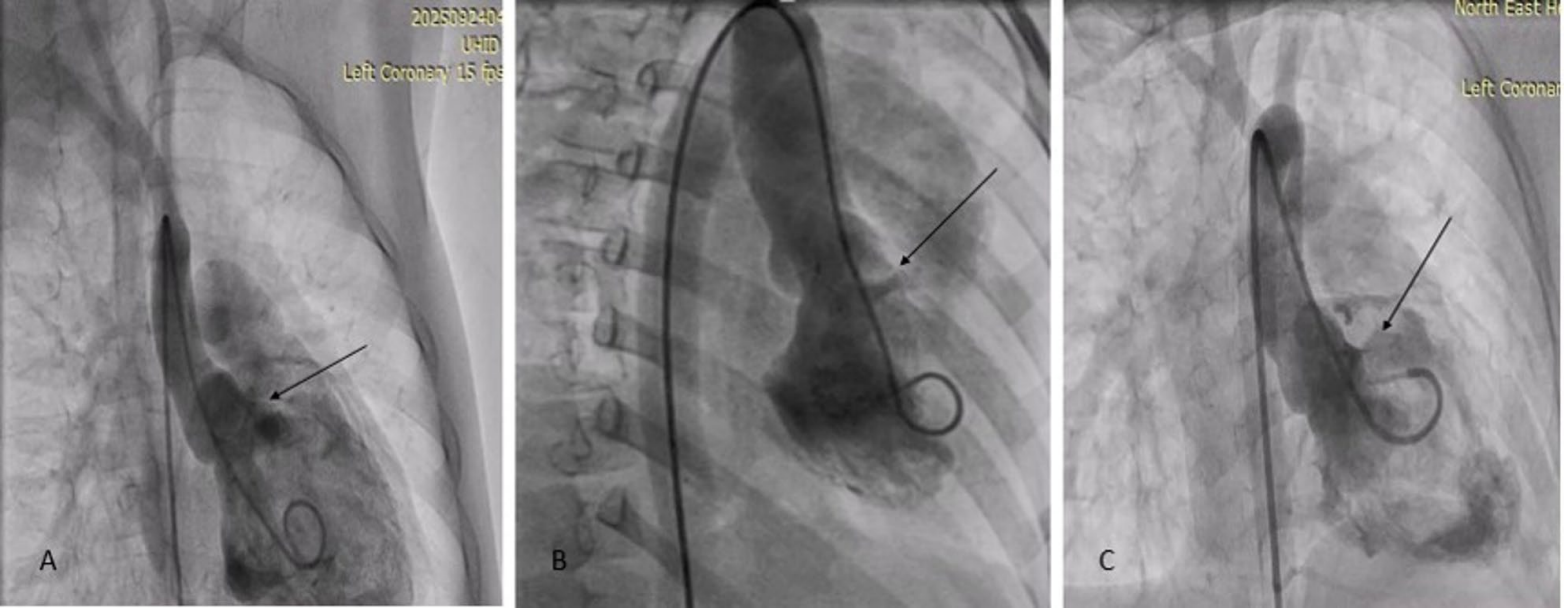
Profiling Outlet and outlet muscular VSD in RAO angiogram (arrow) showing defect in the outlet area

**Fig. 4:**
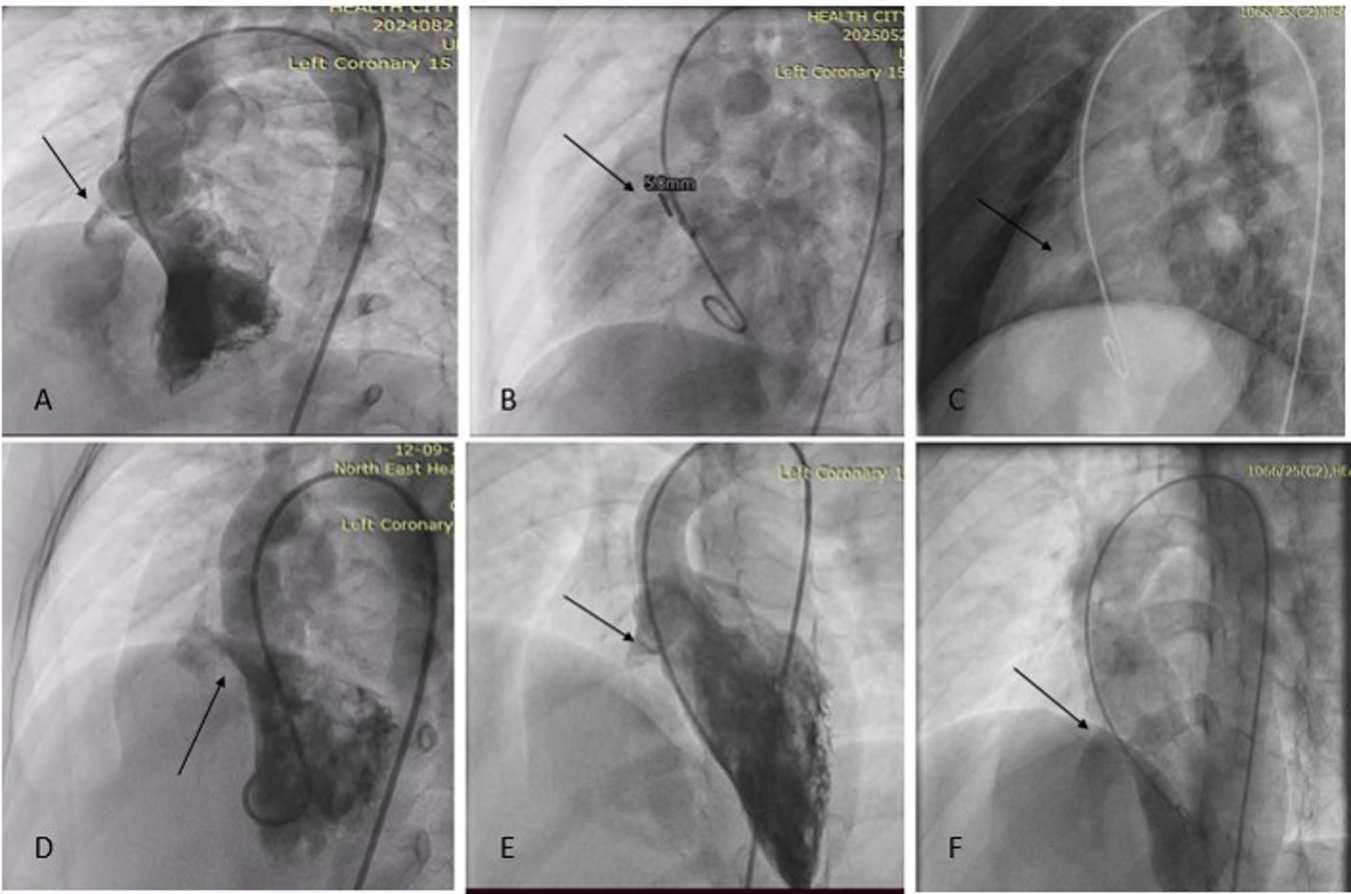
Profiling VSD in various LAO projections with sizing VSD from the RV Side, with an arrow highlighting the defect

**Projections** [3–4]: In all patients, VSD sizing was done after cumulative assessment of echocardiography and LV angiogram. For Outlet VSD and outlet muscular VSD, LV angiogram was obtained in RAO 30^0^ with either Lateral 60^0^ and cranial 20^0^ or Lateral 90^0^. We prefer the Lateral angiogram more for outlet muscular defect and RAO projection in outlet-type defects. Its relationship with the aortic valve, VSD diameter, and AVP and AR severities was then assessed and correlated with echocardiographic findings (Figs. 3 and 4).

**VSD sizing** [5]: The VSD diameters and length were first measured from the echocardiogram; the average of the measurements in at least 2 planes, which must include the parasternal short axis view, was taken. From the left ventricular (LV) angiogram, the most visualized projections during end-systole were taken for measurements and were compared with echocardiographic findings.

**Approach**: We mostly used a retrograde approach for VSD device closure, and for selected cases with larger defects or cases in which it was difficult to advance the delivery sheath, an antegrade approach was used. For the retrograde approach, the VSD was crossed from the LV using either a pigtail catheter (Cordis USA) or 5- or 6-Fr SRC (Cordis, Milpitas, CA, USA), introduced over the 0.035-inch x260 cm J-Tip GLIDEWIRE^®^ (Terumo, Tokyo, Japan). The tip of the catheter was pointed anteriorly to ease crossing the defect. After placing the guide wire in the main pulmonary artery, over the Terumo wire, the VSD delivery sheath or guiding JR was advanced and placed in the RV. For Most cases, we preferred the Amplatzer VSD delivery sheath due to its soft nature and easy movement across aortic cusps.

In the antegrade approach, snaring via the femoral venous catheter to create the arterio-venous (AV) circuit was done. Then 5–8 Fr delivery sheath was advanced from the femoral vein through the circuit and parked in the ascending aorta or LV.

### Device sizing and selection

- Different occlusion devices were used for transcatheter closure as follows: AMPLATZER Duct Occluder II (ADO II; Abbott Medical, MN, USA), KONAR-MF™ Occluder (MFO; Lifetech Scientific, Shenzhen, China), Coccoon VSD occluder (Sahajanad Medicals). Generally, ADO II was used for most OVSD and outlet muscular VSD, especially when the RV exit diameter was ≤ 5.0 mm (depending on device availability on the shelf), whilst for large defects (5.1–8 mm) with torrential flow, KONAR MFO and Cocoon VSD occluder were used. For Outlet VSD -The device, which is 0.5–1 mm larger than the largest RV exit diameter, was used. For outlet Muscular VSD: Devices that were 1–3 mm larger than the largest RV exit diameter were selected. In cases with pulmonary hypertension (PH) and larger muscular defect, device size sizing was upgraded to at least 4–6 mm larger than the RV diameter.
- Type of defect- for outlet, first we preferred low profile ADO II self-centring device.
- Pulmonary hypertension- MFO devices are generally considered.
- Pre-existing AR- ADOII or smaller low-profile MFO devices are usually our first preference.

### On table assessment

**Fig. 5:**
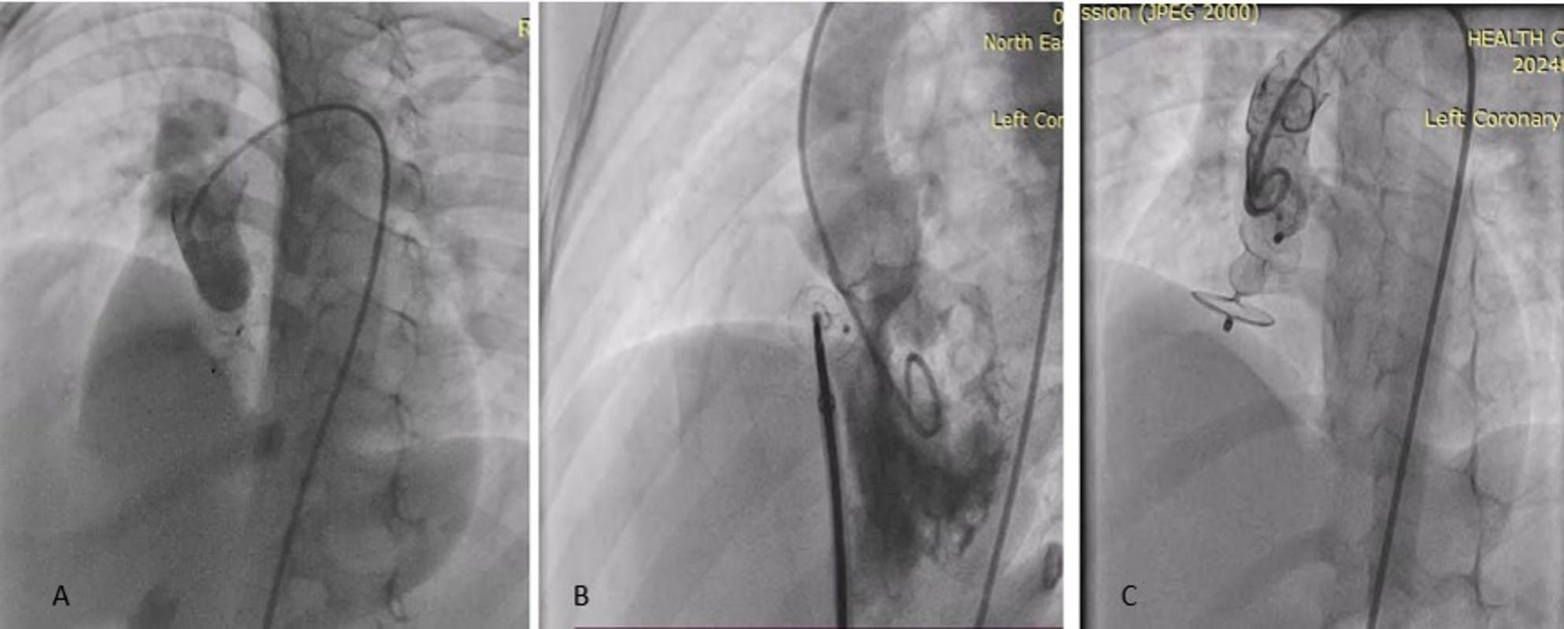
Showing post-deployment angiogram with no aortic regurgitation: **A** aortogram post ADO II implantation, **B** antegrade placement of Cocoon VSD occluder with LV angiogram, **C** Aortogram post implantation of 14/12 KONAR MFO

In case of retrograde approach, hand injection through a long Touhy-Borst (Y connector) attached to the end of the right coronary artery guiding catheter or VSD delivery sheath and the position of the left ventricular disk in relation to the aortic valve cusp was assessed before releasing the device. In case of an antegrade approach, a left ventriculogram or aortic root angiogram was done by pigtail catheter for the same purpose (Fig. 5). Any signs of aortic cusp entrapment during on-table assessment were considered not ideal and were subjected to repositioning, retrieval or resizing.

After device deployment, a thorough assessment was done to see the position of the device, residual defect, any change in the geometry of the aortic valve, impingement of the aortic valve cusp by the disk of the device, appearance of new-onset aortic regurgitation, and any increment in pre-existing aortic regurgitation by transthoracic echocardiography. It was repeated after releasing the device.

### Outcome and follow-up analysis

Favourable outcome: defined as AR≤ mild in severity, an aortic disk of the device not impinging on aortic cusps with no restricted cusp mobility, and absence of significant intra-device shunt or outflow obstruction post procedure.

With no standard definition, it is often difficult to quantify and differentiate between trivial and mild AR. For quantification, uniformity, and to avoid inter-observer bias, we categorize trivial and mild AR degree in the same category as trivial- mild.

Unfavourable outcome: worsening of AR or para-device or significant intra-device shunt or evidence of outflow obstruction.

Follow-up protocol: All cases are followed at 1, 2,6,12 months with echocardiography and ECG, with special emphasis on the aortic valve and AR. Close follow-up after 7–10 days of the procedure was done with cases with complications.

Threshold for re-intervention: moderate or severe AR or significant residual shunt with either symptoms or evidence of LA/LV enlargement (LV Z score > + 2SD for age) on follow-up will be planned for device retrieval and surgical closure.

## Results

- Table 1 summarizes the demographic, echocardiographic, and hemodynamic data for the 21 patients involved in the study. The median age was 56 months (range, 17–278 months), and the median weight was 13.63 kg (range, 8.3–54.5 kg), with a male representation of 62%.
- Most patients were classified as NYHA Class 1, with 43% patients having symptoms of heart failure or volume overload and were receiving anti-failure medications. Of the 21 patients, eight had outlet-type ventricular septal defects (OVSDs), and thirteen had muscular outlet-type VSDs (OMVSDs), with a mean defect size of 3.8 mm, which was larger in the muscular outlet types. Additionally, 66.6% had pre-existing aortic valve prolapse, and five patients exhibited trivial-mild aortic regurgitation before the procedure.

**Table 1:** Demographic and Echocardiographic details of patients

Parameters	Total number of patients (*n* = 21)
Age (months)	56 months
Median	78 months
IQR	Range (17 months to 278 months)
Sex	13 male (62%) 8 female (38%)
Weight (kg)	12.4 kg
Median	23.7 kg
IQR	13.63 ± 5.86
Mean	(Range 8.3–54.5 kg)
NYHA class	12 (class 1) 9 (class 2)
Syndromic	4 (19.04%)
Associated Defects	
Bicuspid Aortic Valve	1
PDA	2
PFO	4
VSD size Median IQR Mean	4 mm 2.9 mm 3.8 mm+/-0.96 mm Range (2.8–7.5 mm) Outlet (2.8–4.6 mm) Outlet muscular (3.8–7.5 mm)
Types of VSD	
Outlet	8
Outlet Muscular	13
Degree of aortic valve prolapse	
1. None	7
2. Mild	11
3. Moderate	3
Degree of pre-existing AR	
None	16
Trivial- Mild	5

In all 21 patients, cardiac catheterisation was performed, with an average Qp: Qs of 1.67 ± 0.43; some degree of pulmonary hypertension (PH) was found among 4 patients (2 with moderate PAH), with a mean pulmonary artery pressure (mPAP) of 18 mmHg. The details of the patient, device, and procedure of the transcatheter cases were summarised in Table 2.

Procedural success was observed in 16 patients (76%), with device implantation failure in 5 patients. Device closure was done in the majority of patients by the retrograde route; the antegrade route was opted for in larger defects and smaller children.

**Fig. 6:**
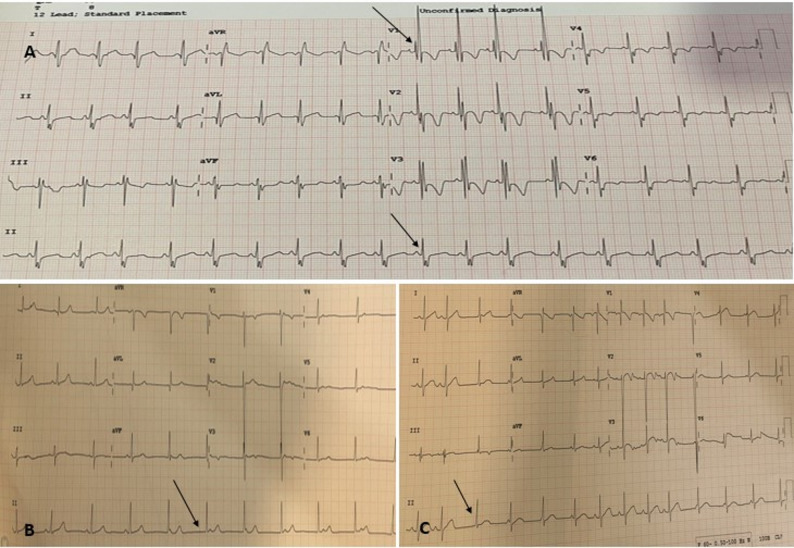
Transient ECG abnormalities on the next day post procedure (**A**-RBBB pattern, **B**-**C** Junctional Rhythm (arrow) with ventricular ectopic), the majority were resolved within 48 h with dexamethasone

- The ADO II device was successfully implanted in 7 cases (43%), the KONAR MFO VSD occluder in 8 (50%), and the Cocoon Muscular VSD occluder was used in 1patient with a mean procedural time of 40 min.
- Among the successful implantations, an increase in the severity of AR was observed in 6/16(37.5%) patients, and an intra-device shunt was observed in 7/16(43.7%) patients on the next day.
- Four out of 16 patients (2 with junctional rhythm, 1 with RBBB, and 1 with ventricular ectopics) (Fig. 6) experienced some electrical abnormalities post-device closure, which were managed conservatively with IV dexamethasone at a dosage of 0.6-1 mg/kg/day for 1–2 days and resolved within 48 h. A notable case demonstrated resolution of junctional rhythm after 10 days of oral doses of prednisolone administered after dexamethasone.
- No LV and RV outflow tract obstruction, hemolysis or device embolization was observed in the present study. There were no major complications infective endocarditis, neurological events, or death) after transcatheter therapy.

**Table 2:** Procedural details

Parameters	Total number of patients
Device closure attempted	21
Qp: Qs (mean)	1.67 ± 0.43 (range 1.3–2.43)
Pulmonary artery pressure (mPAP) (mmHg)	Mean 18+/-0.6 ( Range 9–34 mmHg)
Pulmonary Hypertension(PH) present	4/21 ((19%)
Procedural Success	16 (device implantation) 5 failure cases
Route (*N* = 16) 1. Antegrade 2. Retrograde	5 11
Type of Device Used(*N* = 16)	
1. ADO II	7 (43.7%)
2. MFO VSD Occluder	8 (50%)
3. Cocoon VSD Muscular Occluder	1
Complications (*N* = 16)	
1. Residual shunt (after 24 h)	7 (43.5%)
2. Device embolization	0
3. Increase in the severity of AR(after 24 h)	6(37.5%)
4. Haemolysis	0
5. RV outflow obstruction	0
6. LV outflow obstruction	0
7. Arrhythmias	
• Junctional Rhythm	2
• Ventricular Ectopic	1
• RBBB	1
Mean procedural Time (minutes)	40 ± 16.7

**Fig. 7:**
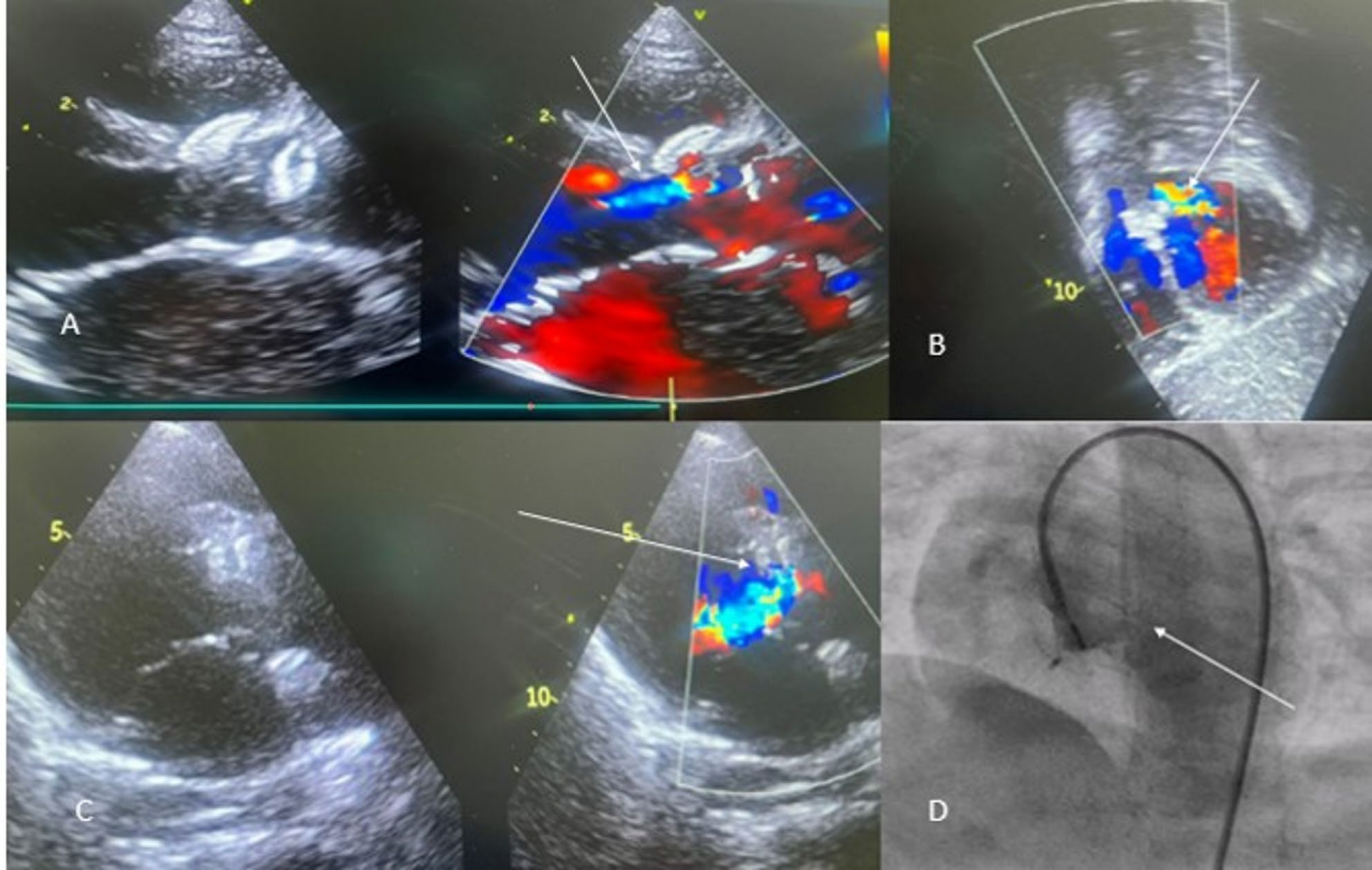
**A**-**C** arrow showing worsening of AR in failure cases after the deployment of the device in echocardiography, **D** depicting an angiogram showing significant AR after device placement (In all cases device was captured and taken out)

The device was not implanted in 5 out of 21 patients: in 3 cases, there was on-table worsening of AR despite downsizing the device; in one case, the patient experienced significant RVOT VT shortly after device deployment; and in one case, the VSD device sheath could not be advanced due to aortic cusp prolapse (Fig. 7). All 5 cases were referred for surgery the next day, with successful outcomes Table 3.

**Table 3:** Detailed analysis of failure cases

Cases	Type of defect	Reason	Device sizing tried
1. 8 years /female/5.6 mm defect	Outlet	An increase in the severity of AR and paradevice flow, hence the device was taken out	7/5, 8/6 MFO
2. 5 years /male 3.3 mm defect	Outlet	Difficulty in the advancement of the VSD delivery sheath	-
3. 2.5 years /male 3.9 mm defect	Outlet	Significant RVOT arrhythmias during the procedure	5/4 ,6/4 ADO II
4. 7 years /male 4.3 mm defect	Outlet Muscular	Worsening of AR on the table	5/6 ,6/4 ADO II
5. 5.5 years /female 7.8 mm defect	Outlet Muscular	Worsening of AR and mild RVOTO	12/10 ,14/12 KONAR MFO

**Table 4:** Outcome and follow-up analysis on the degree of aortic regurgitation and residual shunt with various devices

Parameters	ADO II	KONAR MFO VSD occluder	Cocoon VSD occluder
Cases	9	11	1
Success	7 (77.7%)	8 (72.7%)	1 (100%)
Defect Type			
1. Outlet	4	1	Nil
2. Outlet muscular	3	7	1
Failure cases with worsening of AR on the table	1 (device capture and taken out)	2(device capture and taken out)	Nil
Device Size selection	4/4 − 1	6/4 − 1	8 × 4 -1
	5/4–4	7/5 − 1	
	6/4 − 2	8/6 − 2	
		10/8 − 2	
		12/10 − 1	
		14/12 − 1	
Intra-device Flow			
1. On table	2	6	1
2. The next day (24 h)	2	5	Nil
3. On a 1-year follow-up	1	1	Nil
AR assessment			
A -on the next day (24 h)			
1. None	5	4	1
2. Trivial -Mild	2	4	Nil
3. Moderate	Nil	Nil	Nil
B – on 1-year follow-up			
1. None	4	5	1
2. Trivial -Mild	3	2	Nil
3. Moderate	Nil	1	Nil
C-Increase in severity of AR	1 (None to Trivial-Mild)	1( None to Trivial- Mild) 1(Mild to moderate)	Nil

Table 4 summarizes the outcomes of different devices used.

**Fig. 8:**
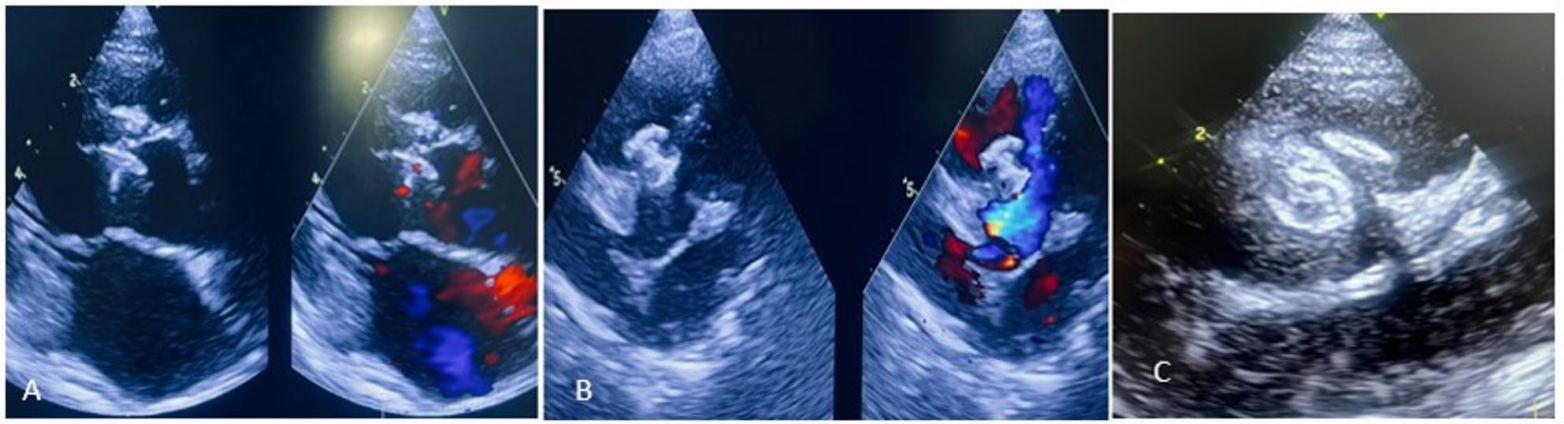
**A**-**C** showing the desirable outcomes after device release on echocardiography with perfect positioning of the device and no significant AR

- ADOII was implanted in 9 patients, achieving a 77.7% success rate. THE KONAR MFO VSD occluder was successfully used in 8 out of 11 patients, while a Cocoon VSD occluder was utilized in 1 case. ADOII was primarily deployed in outlet defects <5 mm, with the common size being 5/4, all using a retrograde approach.
- Two OVSDs required replacement with a smaller ADOII due to the LV disc touching the aortic cusp. One patient experienced a residual shunt with ADOII, which improved after upsizing. KONAR was mainly used for larger outlet muscular VSDs, with 5 patients using sizes like 10/8, 12/10, and 14/12, and 2 needed upsizing for residual shunts. We used Cocoon VSD occluder in 1 patient with an outlet muscular defect with good rims and aortic valve prolapse.5 cases required resizing (3-upsizing and 2- downsizing of devices) were done in the same setting study in our study, but were not considered as separate attempts in our study.
- 7/16 patients had post-procedure intra-device shunt, mostly in the MFO group, with smaller sizes on the next day after the procedure. The majority of this intra-device shunt was not observed in the 1-year follow-up; only 2 patients (1-ADOII and 1 -MFO group) had persistent intra-device shunt, but with no features of haemolysis. Higher residual shunts were reported by HC Lin et al. [11] up to 42% in follow-up.
- Among all 16 patients, a trivial - mild increase in AR severity was observed in 6 patients (37.5%) on the next day of the procedure (2 among the ADOII group, 4 among the MFO group). Follow-up analysis at 1 year showed the severity of AR after transcatheter closure remained unchanged in most patients in both the ADOII and MFO group (81.5%) (Fig. 8). Most patients that had a trivial–mild degree of AR that remained static after the procedure, with no progression on 1-year follow-up, and even regression of AR was noted in 1 patient. Nonetheless, AR increased in 3 cases (18.5%), 2 cases with mild severity (1-ADO II, 1-MFO), and in one case, an increase in moderate severity of AR was seen with the MFO device and is planned for device retrieval and aortic valve repair in future.
- Comparative analysis among 5 cases of pre-existing AR showed failure in 2 cases with worsening of AR on table, and in the remaining 2 cases, the degree of AR remained static on follow-up after the procedure.
- Comparative analysis of different outlet septum VSD showed more favourable outcomes in outlet muscular VSDs than Outlet VSDs (83.6% vs62.5%). Failure rates were higher in outlet VSDs in our study.

## Discussion

Outlet septum VSDs have a higher prevalence in eastern countries, accounting 20% -30% VSD of all cases [6]. The incidence of AVP and AR in the outlet in the literature is reported to be 43–73% and 24–65%, respectively [2, 6–8]. The development of aortic valve prolapse is associated with an increase in aortic regurgitation. Early closure of such defects can prevent further damage to the aortic valve and prevent the progression of aortic regurgitation. Surgery is generally recommended if AVP occurs or the shunt is significant or with the onset of AR, as per AHA guidelines, even in a small VSD. Jung et al [8] reported that 86.4% of patients aged < 4 years who underwent OVSD closure showed AR progression in only 0.98%, which suggests that early closure of OVSD before aortic valve deformity can prevent the development of aortic valve complications. The role of transcatheter closure treatment of these defects remains unclear and controversial [9]. Our study demonstrated that transcatheter closure of these VSDs can be a safer alternative option in selected cases with acceptable rates of outcomes.

**Table 5:** .Comparative analysis with other published studies

Study	Country	Sample size	Mean VSD diameter	Devices	Success rate	Progression of AR	Intra-device shunt on follow-up
H.-C. Lin et al. 2020 [11]	Taiwan	49	4 mm	ADOII	45/49 (91.8%)	9/45 (20%)	19/45(42.2%)
S.roymanee et al. 2022 [12]	Thailand	25	3 mm	ADOII, MFO.APO ,	25/25 (100%)	4/25 (16%)	12%
T.-C. Shyu et al. 2016 [13]	Taiwan	16	3.5	ADO II	14/16 (88%)	2/16 (12.5%)	2/14(12%)
R.B. Kuswiyanto et al.2021 [10]	Indonesia	40	3.1 mm	ADOII, MFO Le VSD Coil	37/40 (92.5%)	11%	6/40(8%)
Present study 2025	India	21	3.8 mm	ADOII, MFO, CocooA muscular VSD Occluder	16/21 (76.1%)	3/16 (18.7%)	2/16(12.5%)

Table 5 presents a comparative analysis with other recently published studies, mostly outlet VSDs. The procedural success rate in the present study was relatively low at 76.1%, likely due to the selection of cases involving larger defect sizes. Additionally, the follow-up analysis showed that the progression of aortic regurgitation (18.7%) and the incidence of intra-device residual shunt (12.5%) were comparable to those reported in other studies. No formal hypothesis testing was performed in our study due to the small sample size. S Romanyee et al. [12] reported complete closure rate was not significantly different between transcatheter and surgical cases, immediately and 12 months after the procedures. (80% and 88% vs. 92% and 96%; *p* > 0.05). New onset or worsening of AR was also not significantly different (*p* = 1)

The main considerations for transcatheter closure of outlet and outlet muscular ventricular defects (VSD) device closure are as follows:

1. **Selection of the case**: Not all cases of outlet and outlet muscular VSD are suitable for transcatheter closure. Inherent complications are more common with a higher degree of aortic valve prolapse, larger defects, PH and smaller weight children. The presence of even small conal muscular tissue can produce good results by the transcatheter route. Thoughtful case selection and a low threshold for surgery should be considered while managing and planning for these cases.
2. **Type of defect** - more favourable results were obtained with outlet muscular defect (11/13) than outlet VSD (5/8) in our study. Selected outlet defects yield good results with low-profile, smaller-sized devices like ADOII and MFO, and can be considered for transcatheter closure. H.C. Lin et al. [11] also reported more favourable results with the muscular outlet type than the outlet type defects.
3. **Sizing and profiling the defect**-the most crucial step for the transcatheter route, with prolapsing of the aortic valve cusp, partially covering the defect; determination of the true defect diameter is challenging. Under-sizing of the defect may cause dislodgement, and over-sizing may interfere with the aortic valve, causing regurgitation. Left ventriculography with RAO angulation, along with more lateral LAO angulation to assess the defect and assessment after delivery sheath crossing inside the defect, gives more information in determining the true size of the defect. Echocardiography may overestimate defect size, whereas angiography often underestimates it due to dynamic aortic cusp movement. Consequently, establishing a correlation between these imaging modalities is essential for accurate assessment.
4. **Selection of the type of device**:As mentioned in our study, device selection can be challenging as these defects often require upsizing and downsizing on the table due to the dynamic aortic cusp. Devices with short lengths are more preferable. In our study, we used three different devices: ADO II is mainly used for smaller defects and mostly outlet types; KONAR MFO and Cocoon VSD occluder were used in larger defects. ADOII are flexible, low-profile, self-centring devices with two flat symmetrical retention discs connected by a central cylindrical centre that can straddle the defect, support the aortic valve leaflets, and can be deployed either antegradely or retrogradely. The limiting factor is that only small defects measuring up to 4–5 mm can be closed. MFO: Wide spectrum. Medium profile device and can be approached from both venous side and aortic side. MFOs are slightly stiffer compared to ADO II, which may interfere with the aortic valve, resulting in regurgitation. Limited studies are available on the use of MFO in the outlet area. M Roy etal [14] reported that if part of the device was still inside aortic cusps with or without causing significant aortic regurgitation, pushing the left ventricular disk with a cable during release of the device helped to keep the device away from aortic cusps. Our study demonstrated successful results with MFO, even with larger devices in the outlet muscular septum, with acceptable results. Cocoon Muscular device: High-profile, double-disk device; can be deployed from both antegrade and retrograde routes. This device is mostly used for defects in the outlet muscular septum with good rims. Limited use of this device has been reported and requires further analysis to assess its safety in the outlet septum.
5. **Size of the devic**e - In our experience, a device 1–2 mm larger than the RV side is sufficient for the majority of outlet defects; however, outlet muscular defects often require a higher size ranging from 2 to 6 mm larger than the RV exit due to the squeezing and dynamic nature of RV outflow, and depending upon age and pulmonary artery pressures, device selection may vary. The muscular outlet can accommodate larger devices without aortic valve damage.
6. **The type of imaging needed during the deployment of the device**: LV/Aorta angiogram and echocardiography are the main tools on the table for assessment during transcatheter closure, and should be meticulously used in these cases. Any severity of more than mild or restricted aortic cusp movement and touching or entrapment of the left disk to the cusp are indications for the retrieval, resizing or repositioning of the device.
7. **Complications and Follow-up management**-

- **Aortic regurgitation**: Worsening of AR was seen in 3 patients, with 2 patients showing an increase that was mild in severity, while only in 1 patient moderate AR severity was observed in follow-up analysis. A similar rate of AR was observed in HC Lin et al. [11] and S. Roymanee et al. [12]. The degree of AR appeared to persist or progress even after surgical closure of VSD, with an incidence ranging between 28 and 63% in different studies. Longer follow-up studies are needed to establish the fate of the aortic valve in transcatheter closure.
- **Intradevice shunt** -In our early stage of transcatheter closure, despite reports of a high incidence of residual shunt (7/16), only two exhibited residual intradevice on 1-year follow-up in the present study. Dynamic AV movement and stretching of the device in the outlet area may cause delayed device endothelialisation in these defects, leading to persistence of residual shunts. Any paradevice residual shunt should be considered abnormal. More incidence of post procedure residual shunt was noted with MFO VSD occluder device in our study, however hemolysis was not reported.
- **Electrical Conduction Abnormalities**: In our case study, 4 patients among the successful cases and 1 among failure cases experienced transient electrical abnormalities, including junctional rhythm and RBBB post-device release, which were not reported in previous studies, which may be due to compression or sheath manoeuvring across the RVOT and were managed successfully with dexamethasone. Various hypotheses have been proposed to account for these irregularities, including the interference of devices with the myocardium, the pressure exerted by the device’s waist and edges on the conduction system during placement, progressive flattening of the device and the induction of inflammatory responses and tissue oedema [15]. Majority may resolve with steroid treatment, such as IV dexamethasone at 0.6-1 mg/kg/day or oral prednisone at 1–2 mg/kg/day, which causes a decrease in inflammation and oedema surrounding conduction tissues. Close follow-up of patients is essential within the first 7 days after the procedure [16].
- **Outflow obstruction**: A notable concern during transcatheter closure is the risk of left ventricular (LV) or right ventricular (RV) outflow obstruction. While some transient flow acceleration may occur post-procedure due to the mechanical device, our study found no reports of outflow obstruction, consistent with other research. Any signs of obstruction should prompt retrieval or repositioning of the device. Additionally, attention must be given to the pulmonary valve regarding potential mechanical pulmonary regurgitation in each case.
- Other major complications, like device embolization A-V block, were not reported in our study.
- Our study demonstrated that while the device interacts with the aortic valve cusp, aortic valve function generally remains intact in the short term following transcatheter treatment. Nonetheless, one patient exhibited progression of aortic regurgitation despite no prior AR with mild cusp prolapse, emphasizing the need for long-term follow-up in such cases.

## Conclusion

Transcatheter closure of outlet and outlet muscular defect in our study demonstrated acceptable rates of short-term outcomes, comparable to previously published transcatheter and surgical series in selected patients and can be feasible and effective as an alternative treatment in selected cases. However method of closure should be tailored on a case-by-case basis. Further long-term evaluation studies are required to assess and confirm the efficacy and safety of this approach, particularly regarding the competency of the aortic valve.

### Study Limitations

This study is a single-centre observational study with a smaller sample. A large sample size and long duration of follow-up are needed to establish the efficacy. Late AR progression beyond 1 year cannot be excluded in our study.

## Electronic Supplementary Material

Below is the link to the electronic supplementary material.

Supplementary file 1

## Data Availability

No datasets were generated or analysed during the current study.

## Abbreviations

VSD: Ventricular septal defect
OVSD: Outlet VSD
OMVSD: Outlet Muscular VSD
AR: Aortic regurgitation
AVP: Aortic valve prolapse
PA: Pulmonary artery
PH: Pulmonary Hypertension
LAO: Left anterior oblique
RAO: Right anterior oblique

## References

[1] Momma K, Toyama K, Takao A, Ando M, Nakazawa M, Hirosawa K et al (1984) Natural history of subarterial infundibular ventricular septal defect. Am Heart J 108:1312e7 6496290 10.1016/0002-8703(84)90759-2

[2] Chung KJ, Manning JA (1974) Ventricular septal defect associated with aortic insufficiency: medical and surgical management. Am Heart J 87:435e8 4274050 10.1016/0002-8703(74)90167-7

[3] Helmcke F, de Souza A, Nanda NC, Villacosta I, Gatewood R et al (1989) Two-dimensional and colour Doppler assessment of ventricular septal defect of congenital origin. Am J Cardiol 63:1112–1116. 10.1016/0002-9149(89)90088-X 2705382 10.1016/0002-9149(89)90088-x

[4] Tatsuno K, Ando M, Takao A, Hatsune K, Konno S (1975) Diagnostic importance of aortography in conal ventricular-septal defect. Am Heart J 89:171–177. 10.1016/0002-8703(75)90042-3 1114944 10.1016/0002-8703(75)90042-3

[5] Ghosh S, Sridhar A, Solomon N, Sivaprakasham M (2018 Jul-Aug) Transcatheter closure of ventricular septal defect in aortic valve prolapse and aortic regurgitation. Indian Heart J 70(4):528–532. 10.1016/j.ihj.2017.11.023 10.1016/j.ihj.2017.11.023 PMC6117845 30170648

[6] Chiu S-N, Wang J-K, Lin M-T, Wu E-T, Lu FL, Chang C-I, Chen Y-S, Chiu I-S, Lue H-C (2005) Mei-Hwan Wu, Aortic Valve Prolapse Associated With Outlet-Type Ventricular Septal Defect, The Annals of Thoracic Surgery. 79(4):1366–1371. 10.1016/j.athoracsur 10.1016/j.athoracsur.2004.10.012 15797078

[7] Jung H, Cho JY, Lee Y (2019) Progression of aortic regurgitation after subarterial ventricular septal defect repair: Optimal timing of the operation. Pediatr Cardiol 40:1696–1702. 10.1007/s00246-019-02206-z 31520096 10.1007/s00246-019-02206-z PMC6848243

[8] Zhang W, Wang C, Liu S, Zhou L, Li J et al (2021) Safety and efficacy of transcatheter occlusion of perimembranous ventricular septal defect with aortic valve prolapse: A six-year follow-up study. Journal ofInterventional Cardiology, 2021, 100081. 10.1155/2021/6634667 10.1155/2021/6634667 PMC7997740 33824626

[9] Kuswiyanto RB, Rahayuningsih SE, Apandi PR, Hilmanto D Muhammad Hasan Bashari, Transcatheter closure of doubly committed subarterial ventricular septal defect: Early to one-year outcome. Int J Cardiol Congenital Heart Disease, 2,2021,100081, ISSN 2666–6685, 10.1016/j.ijcchd.2021.100081

[10] Lin H-C, Lin M-T, Chen C-A, Hsu J-Y, Lin S-M, Wu M-H, Wang J-K Safety and efficacy of transcatheter closure of outlet-type ventricular septal defects in children and adults with Amplatzer Duct Occluder II, Journal of the Formosan Medical Association, 120, Issue 1, Part 1,2021, Pages 180–188, ISSN 0929–6646,10.1016/j.jfma.2020.04.015 10.1016/j.jfma.2020.04.015 32402520

[11] Supaporn Roymanee N, Su-angka W, Promphan K, Wongwaitaweewong J, Jarutach R, Buntharikpornpun P, Prachasilchai Outcomes of Transcatheter Closure in Outlet-Type Ventricular Septal Defect after 1 Year, Congenital Heart Disease, 18, Issue 2,2023, Pages 169–181, ISSN 1747-079X,10.32604/chd.2023.021238

[12] Lin T-CSM-C, Quek Y-W, Lin S-J, Saw H-P, Jan S-L, Fu Y-C Initial experience of transcatheter closure of subarterial VSD with the Amplatzer duct occluder, Journal of the Chinese Medical Association, Volume 80, Issue 8,2017, Pages 487–491, ISSN 1726–4901,10.1016/j.jcma.2016.10.015 10.1016/j.jcma.2016.10.015 28709589

[13] Roy M, Barman S, Gangopadhyay D et al (2025) Transcatheter closure of ventricular septal defect with aortic cusp prolapse with or without mild aortic regurgitation: experience from a tertiary care referral hospital from Eastern India. Cardiol Young 35(9):1889–1898. 10.1017/S1047951125100966 40785400 10.1017/S1047951125100966

[14] Cheung YF, Chiu CS, Yung TC, Chau AK (2002) Impact of preoperative aortic cusp prolapse on long-term outcome after surgical closure of subarterial ventricular septal defect. Ann Thorac Surg 73:622e7 11848094 10.1016/s0003-4975(01)03393-8

[15] Edraki M, Farrokifar M, Amoozgar H, Mehdizadegan N, Mohammadi H et al (2023) Incidence, Risk Factors, and Outcomes of Conduction Disturbances After Percutaneous Closure of Perimembranous Ventricular Septal Defects in Children and Adolescents: A Mid-term Follow-up. Inn J Pediatr 33(5):e135528. 10.5812/ijp-135528

[16] Barone L, Muscoli S, Belli M, Luozzo M, Sergi D, Marchei M, Prandi F, Romana, Uccello, Giuseppe, Romeo, Francesco, Barillà, Francesco (2023) Effect of acute CORticosteroids on conduction defects after Transcatheter Aortic Valve Implantation: the CORTAVI study. Journal of cardiovascular medicine (Hagerstown, Md). 24. 10.2459/JCM.0000000000001516 10.2459/JCM.0000000000001516 37409662

