# Supplementary material for: Feasibility, outcomes and follow-up analysis of transcatheter closure of outlet ventricular septal defect with various devices from North-Eastern India: a single centre observational study
Source: Egypt Heart J. 2026 Feb 27;78:11. doi: 10.1186/s43044-026-00719-6 (PMC12949215; doi:10.1186/s43044-026-00719-6)
Supplement: Supplementary file 1 [file 43044_2026_719_MOESM1_ESM.docx]

| **Parameters** | **ADO II** | **KONAR MFO VSD Occluder** | **Cocoon VSD occluder** |
| --- | --- | --- | --- |
| **Cases** | 9 | 11 | 1 |
| **Success** | 7 (77.7%) | 8 (72.7%) | 1 (100%) |
| **Defect Type**   1. **Outlet** 2. **Outlet muscular** | 4  3 | 1  7 | Nil  1 |
| **Failure cases with worsening of AR on the table** | 1 (device capture and taken out) | 2(device capture and taken out) | Nil |
| **Device Size selection** | 4/4 -1  5/4- 4  6/4- 2 | 6/4- 1  7/5 -1  8/6-2  10/8-2  12/10-1  14/12-1 | 8x4 -1 |
| **Intra-device Flow**   1. **On table** 2. **The next day (24 hours)** 3. **On a 1-year follow-up** | 2  2  1 | 6  5  1 | 1  Nil  Nil |
| **AR assessment**  **A -on the next day (24 hours)**   1. **None** 2. **Trivial -Mild** 3. **Moderate** | 5  2  Nil | 4  4  Nil | 1  Nil  Nil |
| **B – on 1-year follow-up**   1. **None** 2. **Trivial -Mild** 3. **Moderate** | 4  3  Nil | 5  2  1 | 1  Nil  Nil |
| **C-Increase in severity of AR** | 1 (None to Trivial-Mild) | 1( None to Trivial- Mild)  1(Mild to moderate) | Nil |
